# A Smartphone App (AnSim) With Various Types and Forms of Messages Using the Transtheoretical Model for Cardiac Rehabilitation in Patients With Coronary Artery Disease: Development and Usability Study

**DOI:** 10.2196/23285

**Published:** 2021-12-07

**Authors:** Jah Yeon Choi, Ji Bak Kim, Sunki Lee, Seo-Joon Lee, Seung Eon Shin, Se Hyun Park, Eun Jin Park, Woohyeun Kim, Jin Oh Na, Cheol Ung Choi, Seung-Woon Rha, Chang Gyu Park, Hong Seog Seo, Jeonghoon Ahn, Hyun-Ghang Jeong, Eung Ju Kim

**Affiliations:** 1 Cardiovascular Center Korea University Guro Hospital Korea University College of Medicine Seoul Republic of Korea; 2 Division of Cardiology Department of Internal Medicine Hallym University Dongtan Sacred Heart Hospital Dongtan Republic of Korea; 3 Department of Medical Informatics College of Medicine The Catholic University of Korea Seoul Republic of Korea; 4 Sports Medical Center Seoul Republic of Korea; 5 Department of Health Convergence Ewha Womans University Seoul Republic of Korea; 6 Department of Psychiatry Korea University Guro Hospital Korea University College of Medicine Seoul Republic of Korea

**Keywords:** cardiac rehabilitation, smartphone app, coronary heart disease

## Abstract

**Background:**

Despite strong evidence of clinical benefit, cardiac rehabilitation (CR) programs are currently underutilized and smartphone-based CR strategies are thought to address this unmet need. However, data regarding the detailed process of development are scarce.

**Objective:**

This study focused on the development of a smartphone-based, patient-specific, messaging app for patients who have undergone percutaneous coronary intervention (PCI).

**Methods:**

The AnSim app was developed in collaboration with a multidisciplinary team that included cardiologists, psychiatrists, nurses, pharmacists, nutritionists, and rehabilitation doctors and therapists. First, a focus group interview was conducted, and the narratives of the patients were analyzed to identify their needs and preferences. Based on the results, health care experts and clinicians drafted messages into 5 categories: (1) general information regarding cardiovascular health and medications, (2) nutrition, (3) physical activity, (4) destressing, and (5) smoking cessation. In each category, 90 messages were developed according to 3 simplified steps of the transtheoretical model of behavioral change: (1) precontemplation, (2) contemplation and preparation, and (3) action and maintenance. After an internal review and feedback from potential users, a bank of 450 messages was developed.

**Results:**

The focus interview was conducted with 8 patients with PCI within 1 year, and 450 messages, including various forms of multimedia, were developed based on the transtheoretical model of behavioral change in each category. Positive feedback was obtained from the potential users (n=458). The mean Likert scale score was 3.95 (SD 0.39) and 3.91 (SD 0.39) for readability and usefulness, respectively, and several messages were refined based on the feedback. Finally, the patient-specific message delivery system was developed according to the baseline characteristics and stages of behavioral change in each participant.

**Conclusions:**

We developed an app (AnSim), which includes a bank of 450 patient-specific messages, that provides various medical information and CR programs regarding coronary heart disease. The detailed process of multidisciplinary collaboration over the course of the study provides a scientific basis for various medical professionals planning smartphone-based clinical research.

## Introduction

Coronary heart disease (CHD) is a major cause of death [1,2], especially in developing countries [3]. Over the last few decades, there have been many advances in cardiovascular treatment and treatment strategies in patients with atherosclerotic cardiovascular disease, but a residual risk for recurrent cardiovascular events still exists [4]. Various treatment strategies have emerged for secondary prevention, such as optimal medical therapy, including high doses of statins or proprotein convertase subtilisin/kexin type 9 (PCSK9) inhibitors. However, relatively little attention has been paid to lifestyle modification and cardiac rehabilitation (CR).

CR programs deliver comprehensive clinical information, patient support, and monitor patient status. Recent studies have consistently reported the clinical benefits of CR, such as improved survival, reduction of hospital admissions, and improvements in the quality of life [5-7]. Current guidelines strongly recommend CR for secondary prevention [8-10]. However, CR is so underutilized that the participation rate after acute coronary syndrome or revascularization is only 20%-50% [5]. Furthermore, the adherence rate to CR programs at 6 months was only one-third [11]. Although a low referral rate to CR is one of the main factors related to poor participation or adherence, there are several other factors, such as old age, female sex, geographic distance, low physical activity, costs, and lack of insurance coverage, which are difficult or impossible to change [11-14]. Thus, a new model for enhancing the delivery and maintenance of CR services in patients with CHD is required to improve clinical outcomes and reduce social costs.

Recently, several studies have proved the effectiveness of SMS text messages, a simpler form of intervention compared with hospital-based CR, in improving risk factors and patient adherence to treatment [15-17]. Moreover, smartphone apps are expected to be useful tools for CR as they can deliver various forms of content as well as SMS text messages, and in small studies, they have shown favorable clinical results [18,19]. However, previous text messaging systems have many common limitations as follows: (1) The messaging interventions of the previous studies were primarily in a 1-way direction; therefore, the interaction between the patients and medical experts was limited [15]. (2) Although psychosocial factors influence behavioral change, and psychosocial theory–based programs such as transtheoretical model intervention have shown promising results in patients with cardiovascular disease [20,21], it has not been considered during the app development [14]. (3) The majority of messages are text based [22,23], which might have limitations in education and rehabilitation.

This study focused on developing the Application for Self-improvement (AnSim), a smartphone-based, patient-specific messaging app for patients who have undergone percutaneous coronary intervention (PCI), using the transtheoretical model of behavioral change.

## Methods

### Process of Message Development

**Figure 1:**
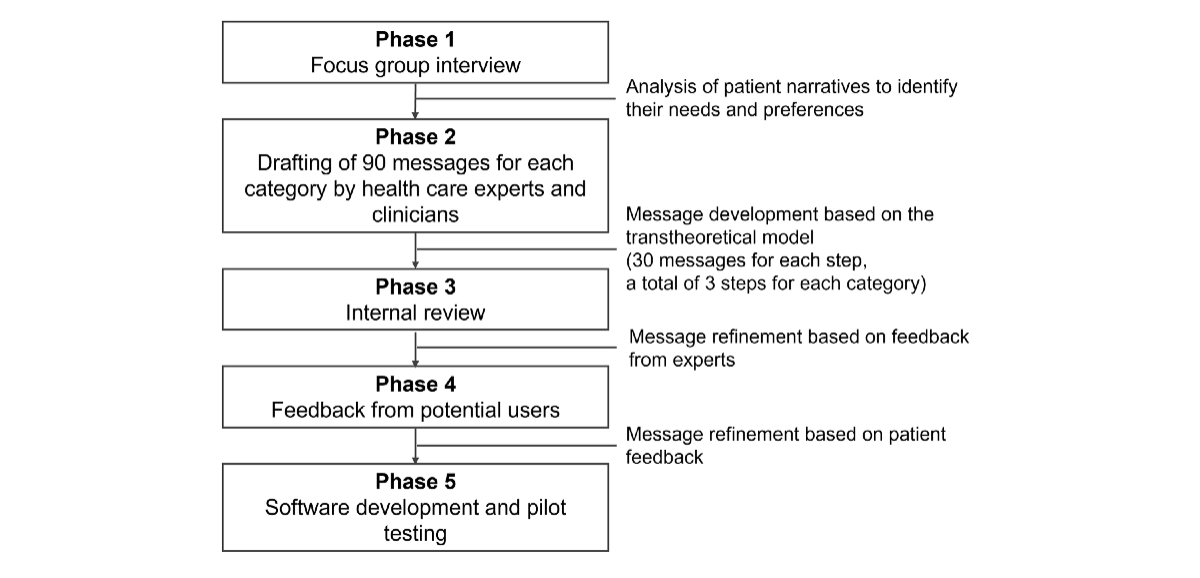
The scheme of the message development process.

A bank of 450 messages was developed by a multidisciplinary team of cardiologists, psychiatrists, nurses, pharmacists, nutritionists, and rehabilitation doctors and therapists using a 5-phase systematic approach. The scheme of the message development process is illustrated in Figure 1.

### Phase 1: Focus Group Interview

**Figure 2:**
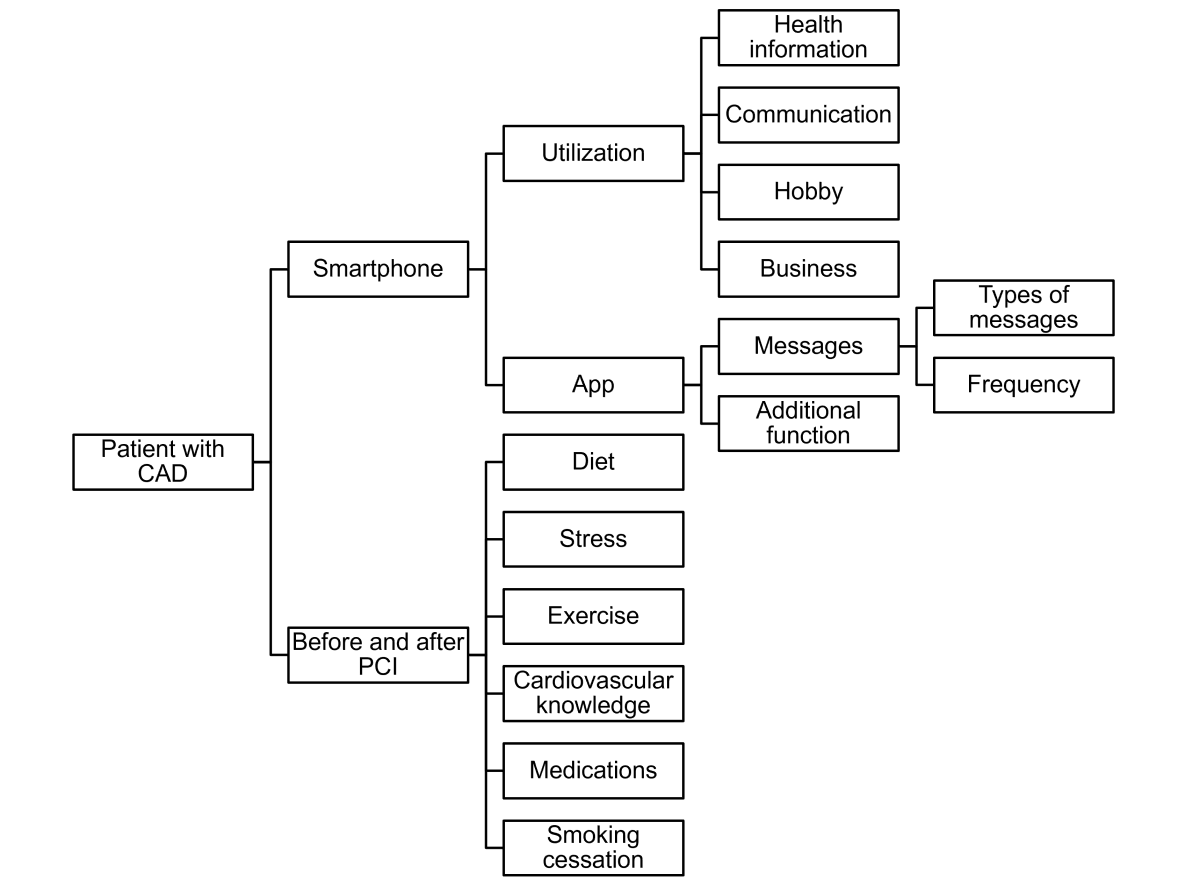
The conceptual framework for the coding. CAD: coronary artery disease; PCI: percutaneous coronary intervention.

A focus group interview was conducted to develop an app that reflects the needs of patients and an understanding of CHD and CR. Patients who had a smartphone and who had undergone PCI within the past 1 year at the Korea University Guro Hospital volunteered for the focus interview. Eight patients of different ages, sexes, and education levels were selected, and in-depth interviews were conducted. The subject of the interview consisted of 5 categories: (1) the degree of smartphone app utilization, (2) exercise, (3) nutrition, (4) stress management, (5) and knowledge about CHD and prevention. The participants of the focus group interview were selected according to the field of CR [24,25] and the design of other CR studies using mobile phone in patients with CHD [15,26,27]. Interviews were recorded with the consent of patients and were transcribed verbatim. Documented interview content was reviewed to exclude repetitive or irrelevant content, such as self-introduction, research participation fee for patients, and personal content to naturally elicit patient’s response. Then, 10 nodes (taking medicine, disease, smoking cessation, first diagnosis, recurrence, nutrition, stress, exercise, smartphone, and app) were derived based on the refined interview contents and a conceptual framework that is widely used for qualitative analysis [28,29] (Figure 2). The interview data were coded under each node and analyzed using NVivo (QSR International), a software package for organizing the analysis of qualitative research.

### Phase 2: Message Development and Its Theoretical Basis

Based on the needs of patients, derived from the focus group interview, experienced health care experts and clinicians drafted messages for the 5 categories: (1) general information regarding cardiovascular health and medications, (2) nutrition, (3) physical activity, (4) destressing, and (5) smoking cessation. Each message was between 40 and 140 Korean characters, in keeping with international guidelines and official educational resources from cardiovascular health–related academic societies.

Furthermore, each message was developed according to 26 behavioral change techniques, which have theoretical backgrounds, such as the information–motivation–behavioral skills model, Theory of Reasoned Action, Theory of Planned Behavior, Social Cognitive Theory, Control Theory, and operant conditioning [30]. In particular, techniques such as the demonstration of behavior and planning of social support, which are difficult to implement with general messaging services, could also be included by providing multimedia, such as sample exercise videos and dietary regimens, and helping participants to connect with smoking cessation centers by actively utilizing smartphone functions. In addition, negative statements in messages were avoided because positive statements are known to help sustainable changes in behavior [31].

To allow participants to receive messages tailored to their stage of behavioral change on a specific topic, messages were developed on the basis of the transtheoretical model of behavioral change, which originally consisted of 5 stages: precontemplation, contemplation, preparation, action, and maintenance [32]. In this study, we simplified these 5 steps into 3 steps: (1) precontemplation, (2) contemplation and preparation, and (3) action and maintenance. According to these 3 simplified steps, behavioral change techniques were categorized, and 30 messages were developed for each step in each category. Finally, a bank of 450 messages was created covering the 5 categories and 3 stages of behavioral change.

### Phases 3 and 4: Internal Review and Feedback From Potential Users

The initially developed messages were checked for evidence, appropriateness, and readability, and then the messages were amended through internal review by experts. Each message was corrected or deleted according to the rating (suitable, need to be corrected, unsuitable) given by 9 researchers during an interdepartmental, internal review process. In the next step, feedback was obtained from potential users (n=458) who were outpatients of various ages, sex, and comorbidities with their CHD being treated at the cardiovascular centers of a secondary general hospital (Sejong General Hospital) and a large tertiary general hospital (Korea University Guro Hospital). Each person evaluated 10 messages and rated the readability and usefulness of each message using a 5-point Likert scale survey questionnaire. Simultaneously, free comments were requested for each message. The messages were reviewed again and refined, based on the feedback from the survey (Multimedia Appendix 1).

### Phase 5: Development of App and Pilot Testing for Message Delivery

Researchers, web designers, and engineers collaborated and developed the user-friendly app by repeatedly discussing the screen composition, function, design, etc. We recruited 20 volunteers to pilot test the message delivery system. All participants were offered brief training at enrollment on how to use the AnSim app and how to input and monitor their health data through the app. Each participant received 6 messages per week for 4 weeks. This frequency was proven to be acceptable for recipients through focus group interviews. Messages were sent randomly at 9 AM, noon, or 3 PM from Monday to Saturday. One message from each of the 5 categories was delivered from the message bank. An additional message was sent for the weak category of each patient.

**Figure 3:**
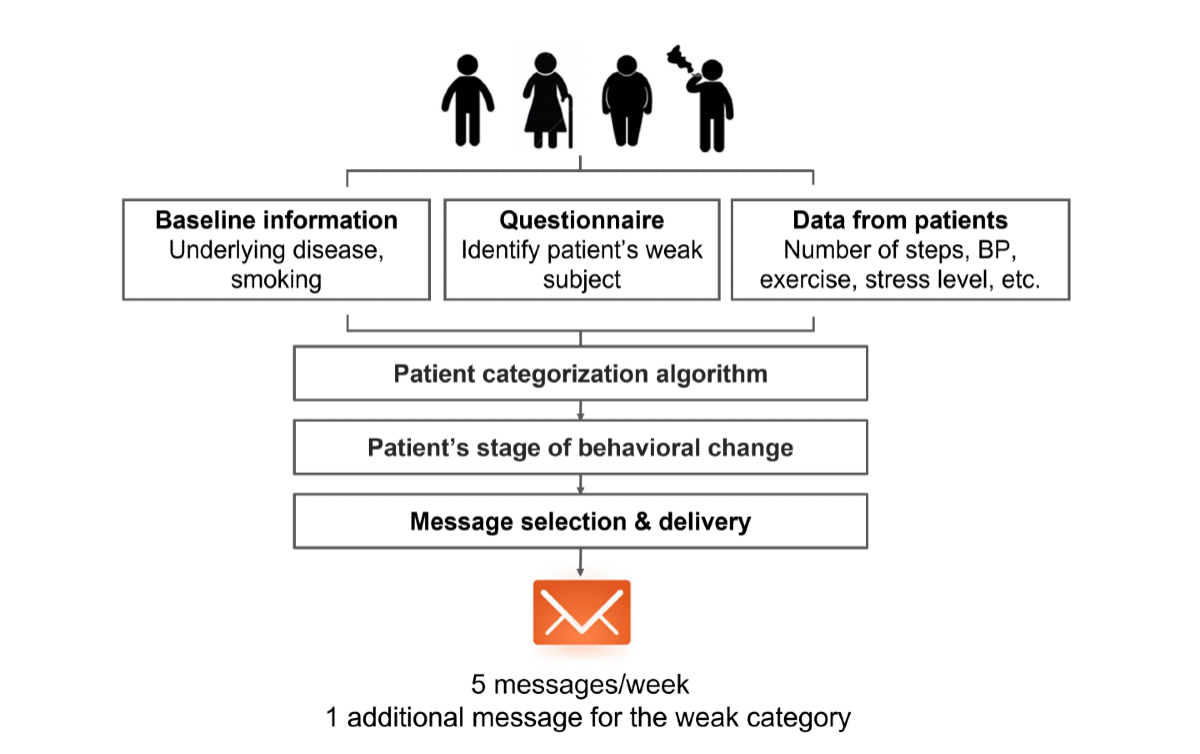
Delivery of patient-specific messages according to baseline characteristics and stage of behavioral change. BP: blood pressure.

To provide patient-tailored messaging intervention, baseline characteristics (ie, having diabetes or not, smoking status) of each participant were identified through the enrollment survey, and messages for the dedicated category were selected randomly by an automated system. For example, participants who were nonsmokers or did not have diabetes did not need to receive messages regarding smoking cessation or diabetes management, respectively. Furthermore, each participant’s behavior stage was identified by administering simple questionnaires each week (Multimedia Appendix 2), and the messages corresponding to a specific stage of behavior change were sent, and no message was repeated (Figure 3). If the message contained video or audio data that could incur additional data costs, a pop-up message preceded the message saying it must be opened in a Wi-Fi environment.

The number of steps taken in a day was automatically recorded by the AnSim app, and the blood pressure, blood glucose, exercise, diet, stress level, and medicine intake were directly recorded by the participants, although it was not enforced. Instead, to enhance patient participation and motivation, a brief weekly review of health data and support messages was sent every week to participants by an independently designated health care provider from an outsourced health care coaching company.

## Results

### Phase 1 and 2: Focus Group Interview and Message Development

The focus group consisted of 8 patients of different ages, sexes, and education levels who had a smartphone and had undergone PCI within the past 1 year. Detailed patient characteristics are presented in Multimedia Appendix 3. In-depth interviews were conducted, and a summary of the results is presented in Textbox 1. Based on the focus group interview, 90 messages in each of the 5 categories were collected: (1) general information regarding cardiovascular health and medications, (2) nutrition, (3) physical activity, (4) destressing, and (5) smoking cessation. Each message was tailored according to 3 stages of behavioral change: (1) precontemplation, (2) contemplation and preparation, and (3) action and maintenance (Table 1).

**Summary of the focus group interview.**

**Utilization of smartphone**

- Participants had no problem in reading and sending SMS text messages regardless of age.
- Participants below 50 years used smartphones not only for communication, but also for information and business. By contrast, participants over 50 years used smartphones for communication only.
- Positive response for receiving messages on cardiac health.

**Exercise**

- Concerns about lacking knowledge about proper exercise.

**Nutrition**

- Participants wanted to know foods and recipes that are good for cardiovascular health.
- Participants tried to avoid fatty foods and eat vegetable-rich diets.

**Stress management**

- Most of the participants did not know about specific and active stress management method.

**Knowledge about coronary artery disease**

- Lack of insight regarding recurrence.
- Lack of knowledge about how to prevent recurrence.

**Table 1:** Examples of messages developed for smoking cessation according to the transtheoretical model of behavior change.

Stage of the transtheoretical model of behavior change	Content	Example (English translation. The original messages were in Korean)
Precontemplation	Provide information about behavior–health link (information–motivation–behavioral skills model)	Smoking is a drug addiction disease, which is registered in the international disease classification.
Contemplation and preparation	Plan social support or social change (social support theories)	Let <NAME>’s family, friends, and co-workers know that you are being treated for heart disease and will quit smoking. In particular, let the friends who smoke know you have heart disease. Everyone will help <NAME> quit smoking.
Action and maintenance	Relapse prevention (Relapse Prevention Theory)	Smoking 1 or 2 cigarettes does not mean that you have failed to quit smoking. Think about the situation in which you smoked, and how you can avoid that particular situation. It may help you in giving up smoking in future.

### Phases 3 and 4: Message Refinement

**Figure 4:**
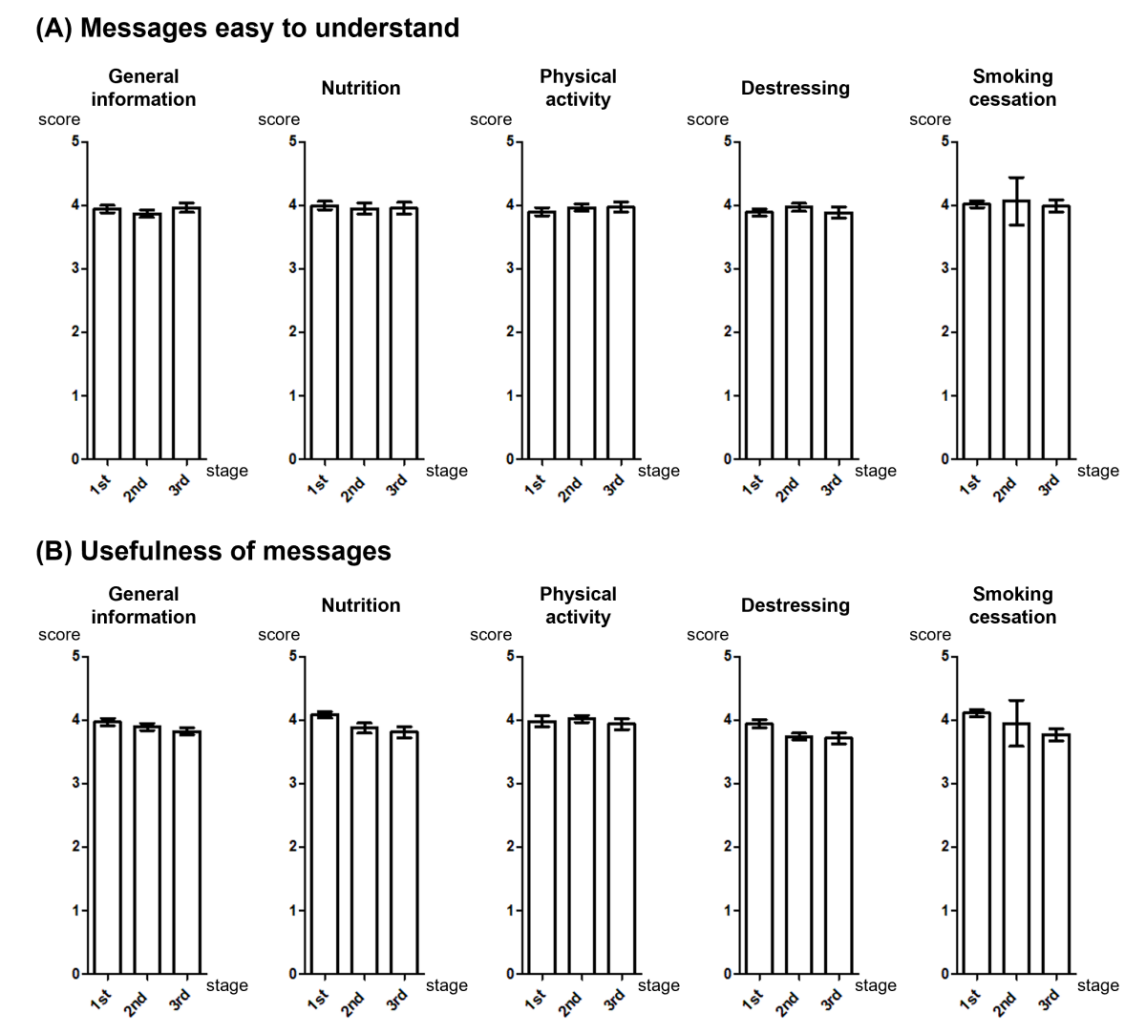
Five point Likert scale scores of various message categories and stages of behavior change.

Message feedback was obtained from potential users (n=458) using a 5-point Likert scale survey questionnaire. Each person evaluated 10 different messages and replied about the readability and usefulness, and approximately 9 comments (feedback) were obtained regarding readability (9.47 responses) and usefulness (9.30 responses) in each message. Nearly 98% of messages received more than 3.0 points regarding readability and usefulness, and the average 5-point Likert scale score was 3.95 and 3.91, respectively (Figure 4). Messages with scores less than 3.5 points were further refined and the final expression was formulated by a linguist. Examples of messages developed after refinement are listed in Table 2.

**Table 2:** Examples of the final version of developed messages after refinement.

Category	Stage of behavior change^a^	Example (English translation. The original messages were in Korean)
General cardiovascular health and medications	2	Taking antiplatelet agents such as aspirin is very important for patients who received percutaneous coronary intervention. When you plan a tooth extraction or endoscopic examination, do not arbitrarily stop the medication without consulting your doctor first.
Nutrition	1	Eating too much salt may burden your heart, leading to swelling and raising the blood pressure (low-salt diet recipes: Link)
Physical activity	3	We applaud you for maintaining a steady routine of exercise. Exercise not only helps you lose weight but also strengthens your heart.
Destress	1	Did you know that depression and coronary artery disease are correlated? Coronary artery disease may lead to depression, and depression in turn can also increase the risk of coronary artery disease.
Smoking cessation	1	Get free smoking cessation counseling. You can get free personalized 1:1 counseling at any time, at home or at work. (Phone number of the national antismoking organization)

^a^1=precontemplation; 2=contemplation and preparation; 3=action and maintenance.

### Phase 5: Development of App and Test of the Delivery System

We developed the AnSim app for both Android and iPhone OS versions. Various multimedia forms, such as exercise videos and dietary regimens, and links for smoking cessation centers have been developed. During the 1-month pilot test, 20 volunteers participated and found no problems in transmitting text and multimedia messages. The participants were evaluated regarding the stage of behavioral change for each category via a simple questionnaire administered through the app (Multimedia Appendix 2) and messages tailored to the current stage were delivered.

## Discussion

### Rationale for Developing the AnSim App

The AnSim app was developed to support behavioral changes and decrease cardiovascular risk factors in patients who had undergone PCI. The messages of AnSim were developed based on the transtheoretical model of behavior change. A 5-phase systematic approach, from focus group interviews to the development of patient-specific message delivery systems, was conducted in collaboration with a multidisciplinary team.

Globally, CHD remains the major cause of death, despite advances in cardiovascular treatment. Further, the incidence rate of CHD is increasing in developing countries [3]. As an integral component of the continuum of cardiovascular care, secondary prevention and CR programs are recommended by most cardiovascular clinical guidelines as a Class I recommendation, and huge amounts of medical resources are devoted toward this endeavor. Although the clinical benefits [5,7] and cost-effectiveness [33] of CR programs have been reported, the supply and accessibility of CR programs are not satisfactory, especially in low-to-middle-income countries [34,35]. There are many hurdles that prevent patients from enrolling into CR programs, such as the distance from the patient’s house to the CR center or a shortage of cost and time. Recently, the use of smartphones has increased worldwide, owing mainly to the development of mobile technology, and interest in mobile health care systems is increasing in various medical fields [14,19]. Smartphone-based CR can be a good alternative strategy that can enhance accessibility to medical care at a low cost [36].

### Comparison With Prior Work

There had been many studies, although with varying number of participants, that showed the feasibility and positive results of mobile phone messaging in reducing body weight [37-39], increasing physical activity [40], and smoking cessation [41]. In particular, the Tobacco, Exercise and Diet Messages (TEXT ME) trial, one of the largest randomized controlled trials involving 710 patients with CHD, demonstrated that the use of an SMS text messaging service resulted in a modest improvement in the management of dyslipidemia and other cardiovascular disease risk factors [15]. These results are not surprising considering that home-based CR programs or education and counseling programs, which do not involve structured exercise therapy, show equivalent CHD prevention effects compared with traditional center-based CR or CR programs, including exercise programs [42].

With regard to smartphone apps, several small randomized studies, including patients with acute coronary syndrome having PCI, demonstrated improvement in treatment adherence [43] and weight loss [44] and a nonsignificant reduction in cardiovascular events [44]. The recent nonrandomized controlled trial with 1064 patients with acute myocardial infarction showed fewer all-cause 30 days readmissions in the digital intervention group compared with the control [45]. Unlike general concerns of smartphone-based interventions for the elderly, who account for a large proportion of patients with CHD, it has successfully improved physical activity and cognitive function in the older population [14,46]. Previous intervention strategies using mobile phones for CR were basically in a 1-way direction, and the contents were provided only as text messages and were not patient specific [8,15,27]. Through the AnSim app, the message is specific, tailored to the patient’s behavioral stage after a brief review of recent medical records. The process is similar to that of a recent randomized controlled trial, the Smartphone and Social Media-Based Cardiac Rehabilitation and Secondary Prevention in China (SMART-CR/SP) trial, involving 312 patients with PCI [26].

### Limitation

The 2-way direction system of the AnSim app is not complete, as it cannot directly answer or react to the patient’s questions and needs immediately. However, the active interaction between CR apps and patients is expected to improve soon as artificial intelligence develops. Instead, the AnSim can deliver patient-specific messages that align with the step of each lifestyle category using the transtheoretical model of behavioral change and serial tracking of the status of patients. In addition, messages in the AnSim app can provide a variety of images, videos, sounds, and feedback, which can improve patient understanding and adherence and may allow for better effects.

### Conclusions

In conclusion, this study reports the development of an app (AnSim) that provides a variety of medical information and CR programs regarding CHD. The messages were developed based on focus interviews, transtheoretical model, feedback, and refinement with various forms of multimedia, and the messages were intended to be specific to baseline characteristics and stage of behavioral change in each participant. Providing CR programs using mobile technology has a huge potential, and we expect that the AnSim app would be helpful for secondary prevention in patients who have undergone PCI. However, future studies are needed to determine the feasibility and efficacy of this app.

## Appendix

Multimedia Appendix 1 The samples of message refinement.

Multimedia Appendix 2 Example of questionnaire for assessing behavioral change steps.

Multimedia Appendix 3 Baseline characteristics of patients participating in the focus group interview.

## Abbreviations

CHD: coronary heart disease
CR: cardiac rehabilitation
PCI: percutaneous coronary intervention
PCSK9: proprotein convertase subtilisin/kexin type 9
