# Supplementary figures and images for: A Smartphone App (AnSim) With Various Types and Forms of Messages Using the Transtheoretical Model for Cardiac Rehabilitation in Patients With Coronary Artery Disease: Development and Usability Study
Source: JMIR Med Inform. 2021 Dec 7;9(12):e23285. doi: 10.2196/23285 (PMC8693185; doi:10.2196/23285)

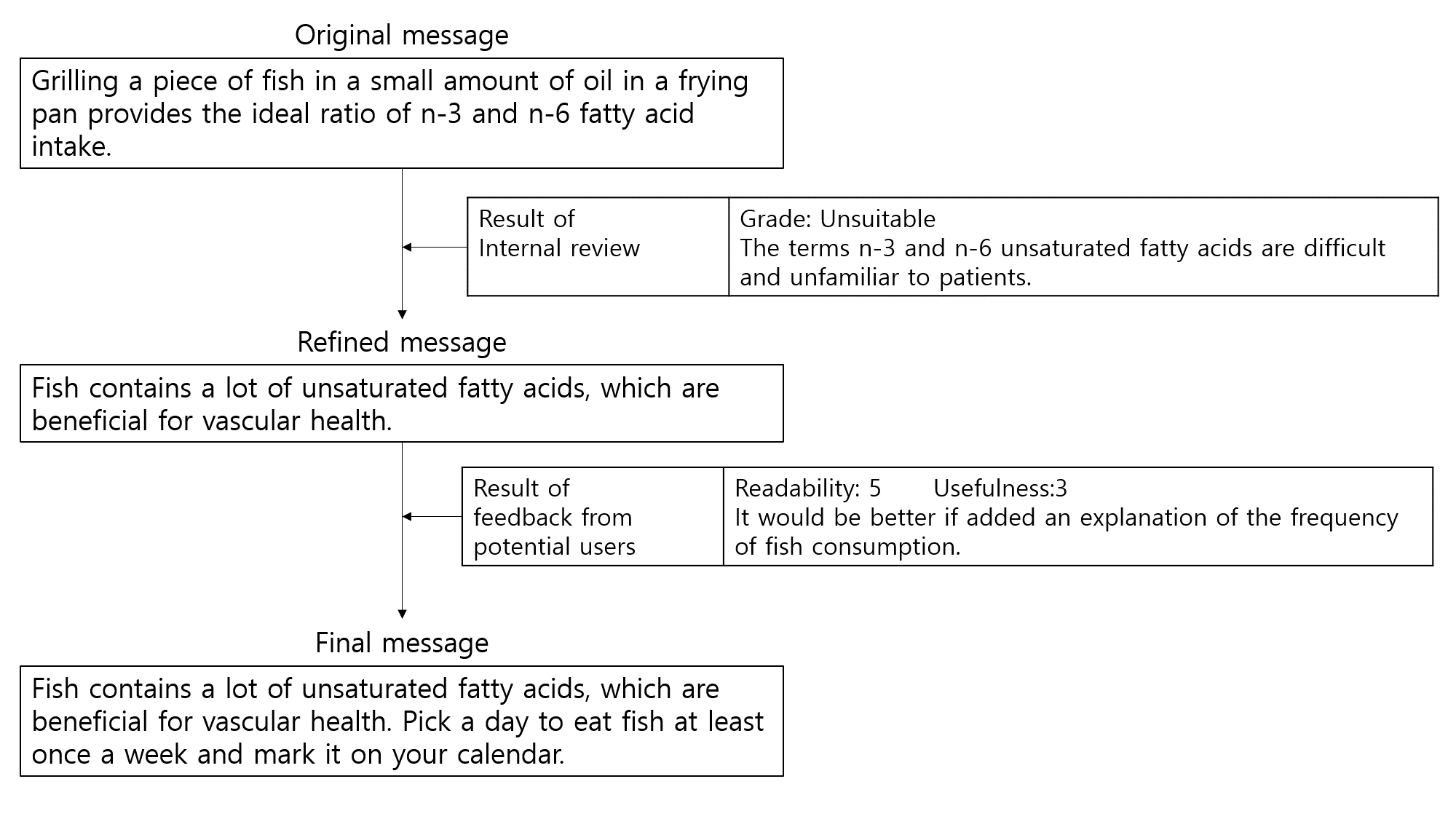

Supplement: Multimedia Appendix 1 [file medinform_v9i12e23285_app1.png]
