# Supplementary material for: A Smartphone App (AnSim) With Various Types and Forms of Messages Using the Transtheoretical Model for Cardiac Rehabilitation in Patients With Coronary Artery Disease: Development and Usability Study
Source: JMIR Med Inform. 2021 Dec 7;9(12):e23285. doi: 10.2196/23285 (PMC8693185; doi:10.2196/23285)
Supplement: Multimedia Appendix 2 [file medinform_v9i12e23285_app2.docx]

**Multimedia Appendix 2. Example of questionnaire for assessing behavioral change steps**

| For general cardiovascular health |  |
| --- | --- |
| I have no plans to start heart health care within six months.  I will start heart health care within six months.  I will start heart health care within 30 days.  I have been steadily improving my heart health within the last six months.  I have been in heart health care for more than six months. | (Yes/No)  (Yes/No) (Yes/No) (Yes/No) (Yes/No) |
