# Supplementary material for: A Smartphone App (AnSim) With Various Types and Forms of Messages Using the Transtheoretical Model for Cardiac Rehabilitation in Patients With Coronary Artery Disease: Development and Usability Study
Source: JMIR Med Inform. 2021 Dec 7;9(12):e23285. doi: 10.2196/23285 (PMC8693185; doi:10.2196/23285)
Supplement: Multimedia Appendix 3 [file medinform_v9i12e23285_app3.docx]

**Multimedia Appendix 3. Baseline Characteristics of Patients Participated in Focus Group Interview**

|  | **Gender** | **Age** | **Initial diagnosis** | **Education** |
| --- | --- | --- | --- | --- |
| 1 | Male | 47 | Stable angina | College graduate |
| 2 | Male | 56 | Stable angina | Middle school graduate |
| 3 | Male | 68 | ST-elevation myocardial infarction | Middle school graduate |
| 4 | Female | 68 | Non-ST-elevation myocardial infarction | High school graduate |
| 5 | Male | 46 | Unstable angina | College graduate |
| 6 | Female | 56 | Non-ST-elevation myocardial infarction | High school graduate |
| 7 | Female | 56 | Unstable angina | High school graduate |
| 8 | Male | 75 | ST-elevation myocardial infarction | Middle school graduate |
